# Inhibition of Anthrax Lethal Toxin-Induced Cytolysis of RAW264.7 Cells by Celastrol

**DOI:** 10.1371/journal.pone.0001421

**Published:** 2008-01-09

**Authors:** Sarah Chapelsky, Sarah Batty, Mia Frost, Jeremy Mogridge

**Affiliations:** Department of Laboratory Medicine and Pathobiology, University of Toronto, Toronto, Ontario, Canada; Research Institute for Children and the Louisiana State University Health Sciences Center, United States of America

## Abstract

**Background:**

*Bacillus anthracis* is the bacterium responsible for causing anthrax. The ability of *B. anthracis* to cause disease is dependent on a secreted virulence factor, lethal toxin, that promotes survival of the bacteria in the host by impairing the immune response. A well-studied effect of lethal toxin is the killing of macrophages, although the molecular mechanisms involved have not been fully characterized.

**Methodology/Principal Findings:**

Here, we demonstrate that celastrol, a quinone methide triterpene derived from a plant extract used in herbal medicine, inhibits lethal toxin-induced death of RAW264.7 murine macrophages. Celastrol did not prevent cleavage of mitogen activated protein kinase kinase 1, a cytosolic target of the toxin, indicating that it did not inhibit the uptake or catalytic activity of lethal toxin. Surprisingly, celastrol conferred almost complete protection when it was added up to 1.5 h after intoxication, indicating that it could rescue cells in the late stages of intoxication. Since the activity of the proteasome has been implicated in intoxication using other pharmacological agents, we tested whether celastrol blocked proteasome activity. We found that celastrol inhibited the proteasome-dependent degradation of proteins in RAW264.7 cells, but only slightly inhibited proteasome-mediated cleavage of fluorogenic substrates *in vitro*. Furthermore, celastrol blocked stimulation of IL-18 processing, indicating that celastrol acted upstream of inflammasome activation.

**Conclusions/Significance:**

This work identifies celastrol as an inhibitor of lethal toxin-mediated macrophage lysis and suggests an inhibitory mechanism involving inhibition of the proteasome pathway.

## Introduction

Anthrax lethal toxin (LeTx) comprises two proteins that are secreted separately by *Bacillus anthracis* and that form complexes on the surface of mammalian cells [1]. The protective antigen (PA) component of the toxin binds host cellular receptors and is proteolytically activated by furin-like proteases. The proteolytic activation of PA causes dissociation of an amino-terminal fragment of the protein, which allows the homo-oligomerization of the receptor-bound carboxy-terminal fragment, PA63. Heptamers of PA63 bind the second toxin component, lethal factor (LF) [2]–[4]. These toxin complexes are internalized by receptor-mediated endocytosis and LF is delivered to the cytosol after translocating through a membrane-spanning pore formed by the heptamer [5], [6]. LF disrupts signaling pathways by cleaving members of the mitogen activated protein kinase kinase (MAPKK) family, thereby interfering with normal cellular functions [7].

The contribution of LeTx to anthrax pathogenesis is complex and is likely mediated through several different mechanisms and cell types [8]. The relative importance to pathogenesis of each of the effects of intoxication is unknown and may differ depending on the site of infection and the infection model. Numerous reports support the notion that the toxin disrupts the immune system, which would aid bacterial survival and support disease progression. LeTx inhibits cytokine expression in T cells, dendritic cells, macrophages, and endothelial cells [9]–[13]. LeTx inhibits production of Type IIA phospholipase A2 by macrophages [14], differentiation of monocytes into macrophages [15], production of immunoglobulin by B cells [16], production of superoxide by neutrophils [17], and impairs neutrophil motility [18]. In addition, LeTx causes cytoxicity in macrophages, dendritic cells and certain types of endothelial cells [19]–[21].

Macrophages seem to both promote and control anthrax infections. Phagocytosis of *B. anthracis* spores by macrophages promotes their germination [22]; but macrophages reduce susceptibility of mice to anthrax infections [23], probably through direct killing of extracellular bacilli. Intoxication of macrophages influences both of these interactions: first, by facilitating the escape of bacilli that have germinated from phagocytosed spores [24], and second, by killing macrophages that can destroy the bacilli.

Macrophages derived from some strains of mice undergo cytolysis after being exposed to LeTx and it was discovered recently that susceptibility to LeTx-induced cytolysis is determined by the allele of the *Nalp1b* gene [25]. Human NALP1 is a component of the inflammasome, which is a complex consisting of NALP1, ASC, caspase-1 and caspase-5 [26]. Assembly of the inflammasome activates caspase-1 and caspase-5, leading to the processing of pro-inflammatory cytokines IL-1β and IL-18. Activation of caspase-1 by murine Nalp1b mediates macrophage cytolysis through the mitochondrial proteins Bnip3 and Bnip3L [25], [27], but it is unclear why the inflammasome is activated by the action of LeTx. LeTx-mediated cytolysis of the susceptible macrophage cell line J774A.1 is accompanied by a proteasome-dependent loss of mitochondrial membrane potential and membrane swelling [28]–[30]. Subsequent to impairment of mitochondria, the plasma membrane is compromised, ATP levels drop, and cytolysis occurs [29].

Celastrol is a small molecule derived from the plant *Triptergium wilfordii* that has been shown to have cytoprotective properties [31], [32], so we sought to determine whether celastrol could protect macrophages from LeTx-induced cytolysis. In this study, we demonstrated that celastrol inhibited LeTx-mediated death of the murine macrophage cell line RAW264.7. Celastrol did not inhibit cleavage of MAPKK1, indicating that it did not block toxin internalization or the proteolytic activity of LF. Furthermore, celastrol was able to protect cells that had been pre-exposed to the toxin, suggesting that it inhibited a late stage of intoxication. We found that celastrol blocked proteasome-mediated destruction of ubiquitylated proteins and prevented LeTx-stimulated processing of IL-18, suggesting that the cytoprotective effects of celastrol are a result of its ability to inhibit the proteasome pathway, thereby preventing inflammasome activation.

## Materials and Methods

### Reagents

Celastrol, rabbit 20S proteasome, and Z-Leu-Leu-Glu-7-amido-4-methylcoumarin (Z-LLE-AMC) were obtained from Calbiochem. MG132, lipopolysaccharide (cat. # L2630), 7-amino-4-methylcoumarine (AMC), and N-succinyl-Leu-Leu-Val-Tyr-7-amido-4-methylcoumarin (Suc-LLVY-AMC) were obtained from Sigma. Glutathione-agarose immobilised GST-S5a, purified 26S proteasome, ATP (cat. # EW9805), t-butoxycarbonyl-Leu-Arg-Arg-7-amido-4-methylcoumarin (Boc-LRR-AMC), and acetyl-L-norleucyl-L-prolyl-L-norleucyl-L-aspartyl-methylcoumarylamide (Ac-nLPnLD-AMC) were obtained from Biomol International. PA and LF were purified as described previously [15].

### Cell culture and cytotoxicity assays

RAW 264.7 cells were maintained in RPMI supplemented with 5% fetal bovine serum and 1% penicillin-streptomycin. For cytotoxicity assays, cells were seeded into tissue culture-treated polystyrene 96-well plates (Corning) at a density of 10^5^ viable cells per well and incubated overnight at 37°C and 5% CO_2_. Cells treated with LeTx were exposed to 10^−8^ M PA and 5×10^−10^ M LF. Cell viability was assessed using the CellTiter 96 Aqueous One Solution Cell Proliferation assay (Promega), as per manufacturer's instructions.

### Western blot assays

Cells were harvested in 1× Cell Lysis Buffer (Cell Signaling Technologies) containing 1 mM phenylmethylsulfonyl fluoride and were sonicated (3×15 seconds). Lysates were cleared by centrifugation; equal amounts of protein were separated on an SDS-polyacrylamide gel and transferred to nitrocellulose. Membranes were blocked in 0.1% Tween-20 TBS (100 mM Tris-HCl pH 8.0, 0.9% NaCl) containing 5% skim milk powder, and were probed with primary antibodies. Antibodies raised against IL-18 (BioVision), IκBα (Santa Cruz Technologies), ubiquitin (Dako), α-tubulin (Sigma), and the N-terminus of MAPKK1 (Upstate Technologies) were used according to manufacturer's instructions.

### GST-S5a agarose pulldown

Cell lysates were prepared from RAW264.7 cells that had been treated with 10 µM MG132 for 1 h. GST or GST-S5a coupled to sepharose (20 µL of 0.5 mg protein/mL resin) was combined with 50 µg lysate in 250 µL of Cell Lysis Buffer (Cell Signaling Technologies) containing 5% glycerol. This mixture was rotated at 4°C for 2 h, either in the presence or absence of 10 µM celastrol. Beads were washed three times with lysis buffer, and proteins were eluted with SDS loading dye. Western blot analysis was performed using anti-ubiquitin antibody.

### 20S proteasome proteolytic acitivity

Reactions were carried out in 50 mM Tris-HCl pH 8.0 containing 0.03% SDS in a volume of 200 µL. Suc-LLVY-AMC was used at a final concentration of 30 µM, and 0.4 µg of 20S proteasome was used per reaction. A constant concentration of 0.4% DMSO was maintained in all wells. Fluorescence (excitation 380 nm, emission 460 nm) was measured every 30 s for 40 min. The slope of the initial linear portion of the curve (over a 15 min interval) was determined using Prism 3.0 (GraphPad Software Inc.). Standard curves were generated using AMC in the presence of the compounds tested, and the amount of AMC liberated per unit time was calculated.

### 26S proteasome proteolytic activity

Reactions were carried out in 50 mM Tris-HCl pH 7.5 containing 40 mM KCl, 5 mM magnesium chloride, 0.5 mM ATP, 1 mM DTT, and 0.5 mg/mL BSA in a volume of 200 µL. Suc-LLVY-AMC, Ac-nLPnLD-AMC, and Boc-LRR-AMC were used at a final concentration of 100 µM. To measure cleavage of Suc-LLVY-AMC, 0.2 µg of 26S proteasome was used per reaction. For the other substrates, 0.75 µg of 26S proteasome was used. A constant concentration of 1% DMSO was maintained in all wells. Fluorescence (excitation 380 nm, emission 460 nm) was measured every 30 s for 2 h. The slope of the initial linear portion of the curve (over a 15 min interval) was determined using Prism 3.0 (GraphPad Software Inc.). Standard curves were generated using AMC in the presence of the compounds tested, and the amount of AMC liberated per unit time was calculated.

### Proteasome proteolytic acitivity of RAW264.7 cell lysates

RAW264.7 cells were washed twice with PBS and suspended in reaction buffer (50 mM Tris-HCl pH 7.5 containing 250 mM sucrose, 5 mM magnesium chloride, 2 mM ATP, 1 mM DTT, and 0.5 mM EDTA). Cells were sonicated for 15 s and lysates were cleared by centrifugation. Reactions were performed in a volume of 200 µL, using a final concentration of 100 µM Suc-LLVY-AMC and 25 µg protein lysate per reaction. A constant concentration of 1% DMSO was maintained in all wells. Fluorescence was measured and data was analyzed as described above for the 26S proteasome assay.

## Results and Discussion

**Figure 1:**
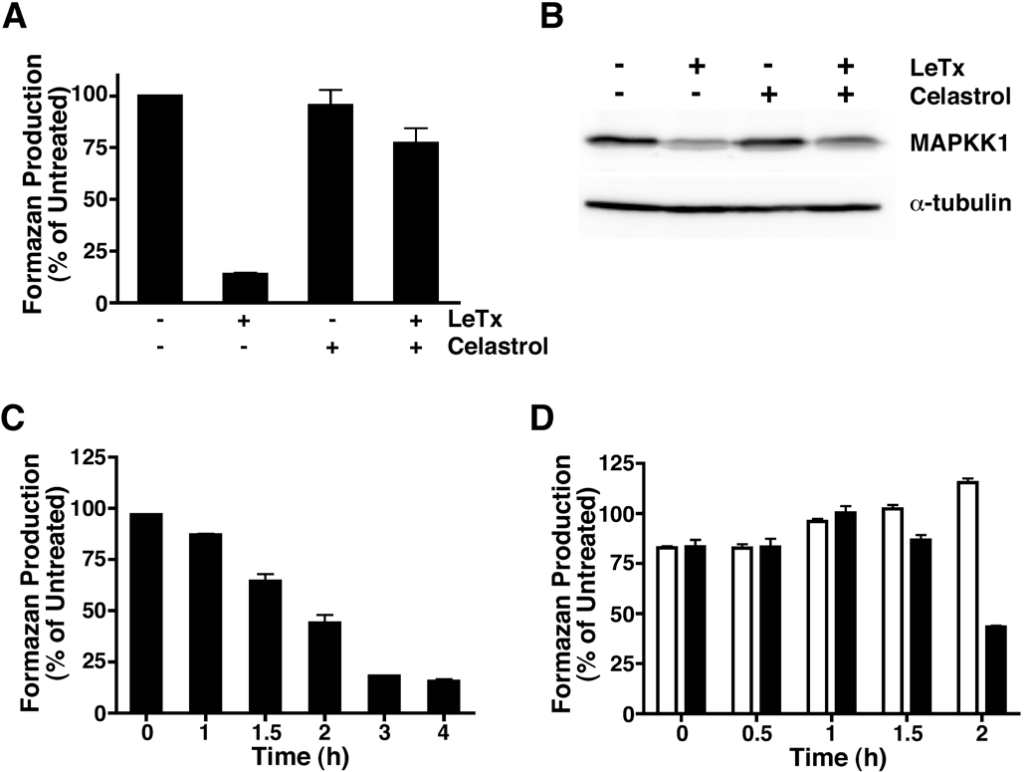
Celastrol blocks LeTx-mediated cytolysis of RAW264.7 cells subsequent to MAPKK1 cleavage. (A) RAW264.7 cells were treated with 3 µM celastrol and/or LeTx (10^−8^ M PA and 5×10^−10^ M LF) as indicated for 4 h. Cell viability was assessed using the MTS assay. The means of three experiments±SEM are reported. (B) RAW264.7 cells were treated with LeTx and/or 10 µM celastrol as indicated for 1 h. Cell lysates were subjected to Western blotting using antibodies that detect MAPKK1 or α-tubulin. A representative result of three independent experiments is shown. (C) RAW264.7 cells were exposed to LeTx for indicated amounts of time and viability was assessed using the MTS assay. The means of three experiments±SEM are reported. (D) Celastrol (10 µM) was added to RAW264.7 cells at the indicated times after cells were treated with LeTx (black bars) at t  =  0 h or were not treated with toxin (white bars). Cell viability was assessed 4 h after LeTx addition by the MTS assay. The means of three experiments±SEM are reported.

To determine whether celastrol can protect RAW264.7 cells from LeTx-induced death, we treated cells for 4 h with LeTx in the absence or presence of celastrol. Cell viability was estimated using the MTS assay, which measures the reduction of a tetrazolium salt to formazan by metabolically active cells. Treatment of cells with LeTx (10^−8^ M PA and 5×10^−10^ M LF) alone reduced metabolic activity, whereas treatment with 3 µM celastrol alone had little effect (Fig. 1A). Cells that were co-treated with LeTx and celastrol exhibited metabolic activity similar to untreated cells. Celastrol also protected the J774A.1 murine macrophage cell line from intoxication (data not shown), demonstrating that this activity is not restricted to RAW264.7 cells.

We next sought to determine whether celastrol inhibited either toxin internalization or the enzymatic activity of LF by monitoring cleavage of MAPKK1, a cytosolic target of LF. Cells were treated with LeTx, celastrol, or both for 1 h. Cellular extracts were prepared and examined by Western blotting using an antibody that recognizes the amino terminus of MAPKK1; LF cleaves an amino-terminal segment from MAPKK1, so this antibody does not detect the cleaved protein. A reduced amount of full-length MAPKK1 was observed in cells treated with LeTx compared to untreated cells (Fig. 1B). Celastrol did not prevent cleavage of MAPPK1 by LeTx, however, indicating that celastrol did not inhibit the delivery of LF to the cytosol or the proteolytic activity of this enzyme.

Since celastrol did not inhibit substrate cleavage by LF, this result indicated that this compound might block a late step in intoxication. To address this possibility, we performed a time-course assay to determine how late during intoxication celastrol could confer protection (Figs. 1C, D). Celastrol was added to cells at different times after toxin addition and the MTS assay was performed 4 h after the toxin was added. In parallel, cells were treated for different amounts of time with LeTx alone to assess the metabolic activity of cells at the times of celastrol addition. A slight reduction in metabolic activity was observed after 1 h of toxin treatment, which became more pronounced at 1.5 h and 2 h (Fig. 1C). By 3 h of toxin treatment, an almost maximal reduction in metabolic activity was observed. Remarkably, 10 µM celastrol was able to completely protect cells that had been exposed to LeTx for 1.5 h and stopped further loss in metabolic activity in cells exposed to toxin for 2 h (Figs. 1C, D). These results indicate that celastrol protects cells by inhibiting a process that occurs late in intoxication.

**Figure 2:**
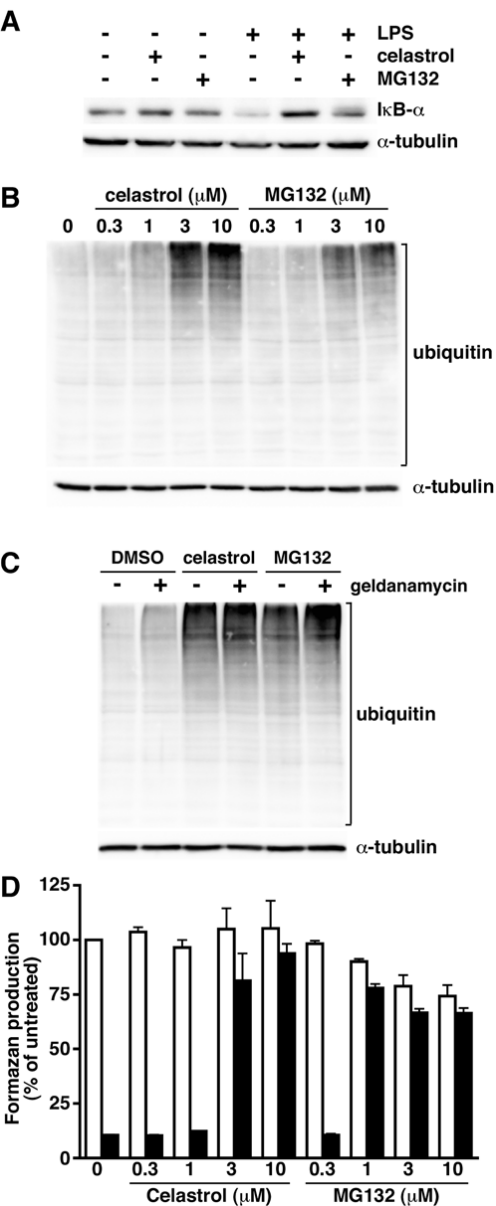
Celastrol inhibits the proteasome-dependent degradation of proteins. (A) RAW264.7 cells were treated with 3 µM celastrol or 10 µM MG132 (or DMSO vehicle) for 30 min, followed by a 10 min treatment with 1 µg/mL LPS. Cellular lysates were prepared and probed for IκB-α or α-tubulin by Western blotting. A representative result of three independent experiments is shown. (B) RAW264.7 cells were treated with indicated concentrations of celastrol or MG132 for 1 h. Cellular lysates were prepared and probed with anti-ubiquitin or anti-α-tubulin. A representative result of three independent experiments is shown. (C) RAW264.7 cells were treated with 10 µM celastrol, 10 µM MG132 and/or 10 µM geldanamycin for 1 h. Cellular lysates were prepared and probed with anti-ubiquitin or anti-α-tubulin. A representative result of three independent experiments is shown. (D) RAW264.7 cells were treated with various concentrations of celastrol or MG132 in the presence (black bars) or absence (white bars) of LeTx for 4 h. Cell viability was assessed using the MTS assay. The means of three experiments±SEM are reported.

Since celastrol has been reported to inhibit proteasome activity in prostate cancer cell lines [33], we assessed whether celastrol might inhibit the proteasome-dependent degradation of proteins in RAW264.7 cells. To test this idea, we treated RAW264.7 cells with lipopolysaccharide (LPS) and probed cell lysates for IκB-α, which is degraded by the proteasome in cells that have been stimulated by LPS. Western blotting indicated that LPS treatment led to the degradation of IκB-α and that this could be inhibited by the proteasome inhibitor, MG132 (Fig. 2A). Celastrol was also able to inhibit the degradation of IκB-α at a concentration that protected RAW264.7 cells from LeTx. As an additional test of whether celastrol inhibited the degradation of ubiquitylated proteins, we treated cells with either MG132 or celastrol and examined cell lysates for ubiquitylated proteins by Western blotting with an anti-ubiquitin antibody (Fig. 2B). We observed that treatment of cells with either MG132 or celastrol led to the accumulation of ubiquitylated proteins, suggesting that celastrol inhibits a step in the proteasome pathway. Interestingly, celastrol treatment resulted in the accumulation of more ubiquitylated proteins than did MG132. Because MG132 is a potent inhibitor of the proteasome, we speculated that the ability of celastrol to cause a greater accumulation of ubiquitylated proteins than MG132 might be a result of an additional activity of celastrol that induces the accumulation of misfolded proteins. Indeed, celastrol has been reported to inhibit HSP90 [34]; inhibition of HSP90 would cause an accumulation of misfolded proteins that would subsequently be ubiquitylated. To test this hypothesis, we treated cells with either celastrol or MG132 in the presence or absence of the HSP90 inhibitor geldanamycin (Fig. 2C). The combination of geldanamycin and celastrol resulted in a level of ubiquitylated proteins similar to that in cells treated with celastrol alone. In contrast, geldanamycin increased the level of ubiquitylated proteins in cells treated with MG132. These results indicate that the higher level of ubiquitylated proteins in celastrol-treated cells compared to MG132-treated cells results from the ability of celastrol to induce protein misfolding (through the inhibition of HSP90) in addition to inhibiting proteasome-mediated degradation of proteins.

We next assessed whether a threshold of total cellular ubiquitylated proteins was required to protect RAW264.7 cells from LeTx. Cells were treated with LeTx in the presence of various concentrations of either celastrol or MG132 and the cells were assessed for viability. Celastrol protected cells at a concentration of 3 µM, but not at 1 µM; MG132 protected cells at 1 µM, but not at 0.3 µM (Fig. 2D). Because there are slightly more ubiquitylated proteins in cells treated with 1 µM celastrol than in cells treated with 1 µM MG132 (Fig. 2B), we conclude that there is not a direct correlation between the bulk accumulation of ubiquitylated proteins and the protection of cells from intoxication. It is possible that inhibiting the degradation of only one or a few ubiquitylated proteins prevents LeTx-mediated cytolysis.

**Figure 3:**
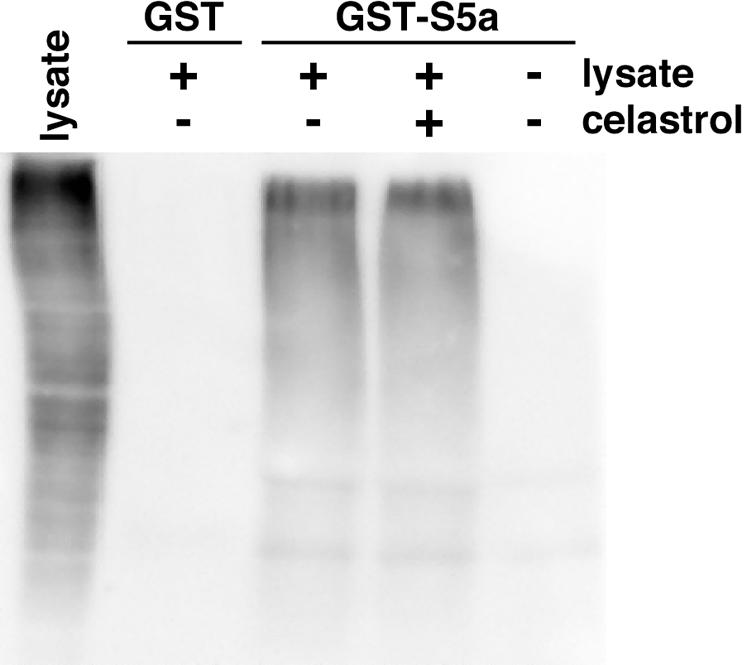
Celastrol does not inhibit the interaction between ubiquitylated proteins and S5a. Cellular lysates were incubated with either GST or GST-S5a coupled to glutathione-sepharose in the presence or absence of celastrol, as indicated. Associated proteins were precipitated, subjected to SDS-PAGE and probed for ubiquitin by Western blotting. A representative result of three independent experiments is shown.

Ubistatins are compounds that block proteasome-mediated protein degradation by binding ubiquitin chains, thereby inhibiting the interaction between ubiquitylated proteins and the proteasome [35]. To determine if celastrol functions similarly, we assessed whether celastrol blocked the ability of the ubiquitin receptor S5a to bind ubiquitylated proteins. Glutathione-S-transferase (GST) or a GST-S5a fusion protein was attached to glutathione-sepharose beads and the beads were mixed with RAW264.7 cellular lysates and then centrifuged to isolate associated proteins. The associated proteins were electrophoresed on denaturing polyacrylamide gels and then probed with anti-ubiquitin antibody (Fig. 3). GST-S5a precipitated ubiquitylated proteins, whereas GST did not. Celastrol did not prevent GST-S5a from binding ubiquitylated proteins, suggesting that celastrol does not interfere with the binding of ubiquitin chains to the proteasome.

**Figure 4:**
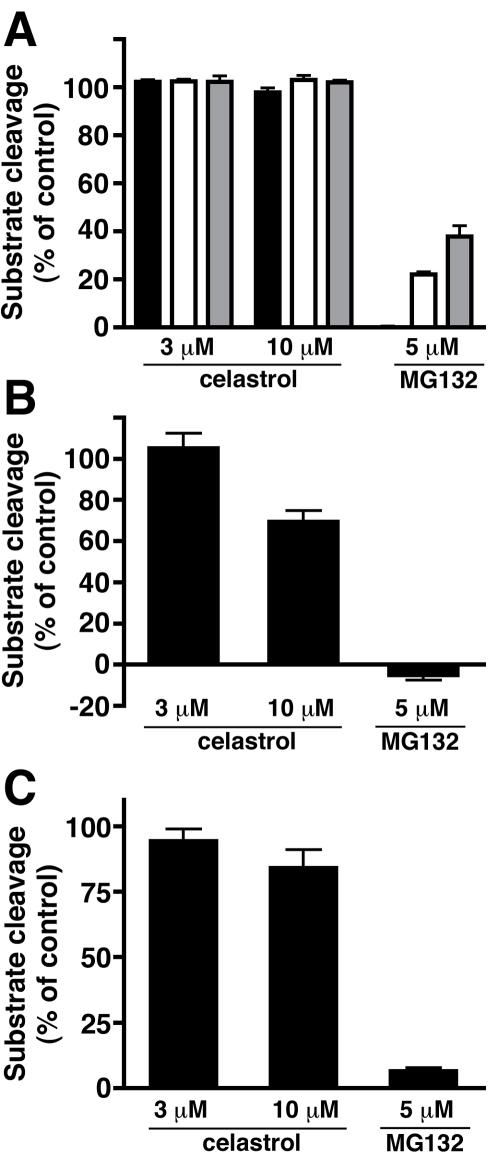
Celastrol is not a potent inhibitor of the proteasome *in vitro*. (A) The chymotrypsin-like (black bars), caspase-like (white bars), and trypsin-like (grey bars) activities of purified 26S proteasome were assessed in the presence or absence of celastrol or MG132. (B) The chymotrypsin-like activity of purified 20S proteasome was assessed in the presence or absence of celastrol or MG132. (C) The chymotrypsin-like activity of the proteasome in RAW264.7 cell lysate was assessed in the presence or absence of celastrol or MG132. Values represent the means of three experiments±SEM.

We next assessed whether celastrol inhibited the proteolytic activity of the proteasome. The 26S proteasome is a large multi-subunit complex consisting of a 20S barrel-shaped core complex and two 19S regulatory complexes [36]. The regulatory complexes ensure that only ubiquitylated proteins access the inside of the barrel where the proteolytic sites reside. The proteasome exhibits three distinct proteolytic activities, chymotrypsin-like, trypsin-like, and caspase-like, which can be assayed individually using fluorogenic substrates [37]. The chymotrypsin-like site is thought to be the most important of the three, although the trypsin-like and caspase-like activities must also be inhibited to reduce the degradation of most proteins by 50% [37]. We mixed purified 26S proteosome with the fluorogenic substrate N-succinyl-Leu-Leu-Val-Tyr-7-amido-4-methylcoumarin (Suc-LLVY-AMC) to measure chymotrypsin-like activity; t-butoxycarbonyl-Leu-Arg-Arg-7-amido-4-methylcoumarin (Boc-LRR-AMC) to measure trypsin-like activity; or acetyl-L-norleucyl-L-prolyl-L-norleucyl-L-aspartyl-methylcoumarylamide (Ac-nLPnLD-AMC) to measure caspase-like activity. Whereas MG132 inhibited cleavage of the three substrates, neither 3 µM nor 10 µM celastrol was able to inhibit cleavage of the substrates by the 26S proteasome (Fig. 4A). Celastrol showed some inhibitory activity against the chymotryptic acivity of the 20S proteasome at 10 µM, but not at 3 µM (Fig. 4B), a concentration that was sufficient to cause the accumulation of ubiquitylated proteins *in vivo* (Fig. 2B). To test further whether celastrol inhibited proteasome activity, we incubated RAW264.7 cell lysates with Suc-LLVY-AMC. MG132 reduced the rate of cleavage of this substrate, but celastrol exhibited little inhibitory activity (Fig. 4C).

**Figure 5:**
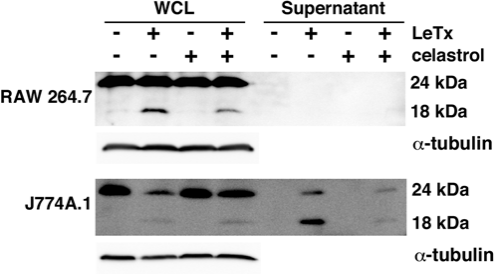
Celastrol prevents LeTx-mediated activation of IL-18 processing. RAW264.7 or J774A.1 cells were treated for 2 h with LeTx (10^−8 ^M PA, 5×10^−10^ M LF) in the absence or presence of 3 µΜ celastrol, as indicated. Cellular lysates were prepared and probed for IL-18 and α-tubulin by Western blotting. Cellular supernatants were collected and probed for IL-18 by Western blotting. Representative results of three independent experiments are shown.

Since it has been reported previously that inhibition of proteasome activity prevents activation of the Nalp1b inflammasome [38], [39], we sought to determine whether celastrol inhibits processing of IL-18 by the Nalp1b inflammasome. RAW264.7 cells were incubated with LeTx in the absence or presence of celastrol for 2 h and cellular lysates and supernatants were subjected to Western blotting using an antibody raised against IL-18. The 18 kDa mature form of IL-18 was observed in lysates derived from cells treated with LeTx and a reduced amount was present in lysates derived from cells treated with both LeTx and celastrol; no IL-18 was detected in the cell supernatants in these conditions (Fig. 5). J774A.1 cells that were treated with LeTx had a reduced amount of the 24 kDa form of IL-18 in cell lysates; the processed form was observed in the supernatants. The stimulation of IL-18 processing by LeTx was reduced in cells that were co-treated with celastrol (Fig. 5). These results are consistent with celastrol inhibiting an intoxication step upstream of Nalp1b inflammasome activation.

In summary, we have demonstrated that celastrol inhibits LeTx-induced cytolysis of RAW264.7 murine macrophage cells. The concentrations required to protect cells (Fig. 2D) were similar to the concentrations that led to the accumulation of ubiquitylated proteins (Fig. 2B). Comparison of celastrol with MG132, however, suggested that protection from cytolysis did not correlate with a threshold level of total ubiquitylated proteins – this suggests that the degradation of a relatively small subset of ubiquitylated proteins may be required for cytolysis. The spectrum of ubiquitylated proteins likely differs between celastrol-treated and MG132-treated cells because celastrol inhibits HSP90, which helps to fold a variety of proteins, in addition to inhibiting proteasome-mediated degradation of proteins. Inhibition of HSP90 by geldanamycin did not protect cells from LeTx (data not shown).

Proteasome activity has previously been implicated in LeTx-mediated cytolysis by structurally unrelated proteasome inhibitors [28]. Recently, a report suggested that celastrol inhibits 20S proteasome activity [33]. Using a fluorogenic substrate, we did detect a slight decrease in 20S proteasome activity *in vitro*, but not in 26S proteasome activity – although 20S proteasome does exist in cells, it is thought that the 26S proteasome is responsible for the degradation of ubiquitylated proteins [37]. Thus, we believe that celastrol either inhibits the proteolytic activity of the 26S proteasome only in intact cells, or it inhibits another step in the ubiquitin-proteasome pathway. Inhibition of the ubiquitin-proteasome pathway is a potential therapeutic strategy for the treatment of cancer and neurodegenerative diseases [40], so it will be of interest to characterize further how celastrol inhibits this pathway and to determine whether it could be used to treat anthrax.
